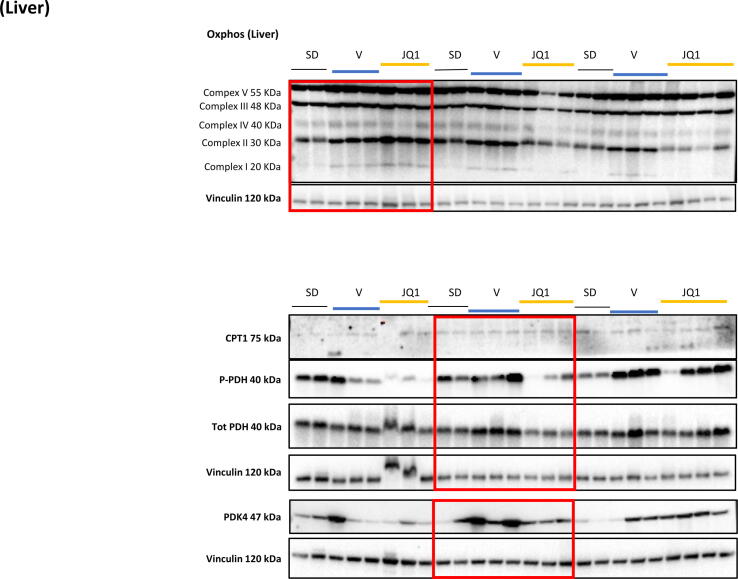# The BET inhibitor JQ1 targets fat metabolism and counteracts obesity

**DOI:** 10.1016/j.jare.2024.02.001

**Published:** 2024-02-15

**Authors:** Claudia Fornelli, Alessia Sofia Cento, Lorenzo Nevi, Raffaella Mastrocola, Gustavo Ferreira Alves, Giuseppina Caretti, Massimo Collino, Fabio Penna

**Affiliations:** a Department of Clinical and Biological Sciences, University of Torino, Turin, Italy; b Department of Biosciences, University of Milano, Milan, Italy; c Department of Neurosciences “Rita Levi Montalcini”, University of Torino, Turin, Italy

**Keywords:** Obesity, Sarcopenic obesity, JQ1, Adipose tissue, Lipid metabolism, Lipolysis

## Abstract

**Introduction:**

Obesity, one of the most frequent health problems in the adult population, is a condition characterized by excessive white adipose tissue accumulation and accompanied by the increased risk to develop other disorders such as type II diabetes, cardiovascular disorders, physical disability, frailty and sarcopenia. Total fat mass frequently increases during aging, often coexisting with sarcopenia, thus resulting in an emerging condition defined sarcopenic obesity (SO). Our previous data demonstrated the relevant role of the bromo and extra-terminal domain (BET) proteins inhibitor JQ1 in attenuating inflammation and fibrosis in sarcopenic mice. Moreover, we preliminarily observed that JQ1 administration markedly reduces white adipose tissue mass, suggesting a potential role of BET proteins on visceral fat deposition during aging.

**Objectives:**

Starting from those observations, the aim of this study was to investigate the ability of JQ1 to reduce adiposity in a chronic diet-induced obesity (DIO) mouse model mimicking the human metabolic syndrome.

**Methods:**

Male C57BL/6J mice were divided in subgroups, either fed a standard diet or a high fat diet for 22 or 12 weeks, treated over the last 14 days with JQ1 or with vehicle.

**Results:**

The results showed that JQ1 administration reduces fat mass, preserving skeletal muscle mass and function. A direct JQ1 lipolytic effect was demonstrated on mature adipocyte cultures. JQ1-mediated loss of adipose tissue mass was not associated with systemic inflammation or with lipid accumulation in muscle and liver. JQ1 administration did not impinge on skeletal muscle metabolism and oxidative capability, as shown by the lack of significant impact on mitochondrial mass and biogenesis.

**Conclusion:**

In conclusion, the current data highlight a potential benefit of JQ1 administration to counteract obesity, suggesting epigenetic modulation as a prospective target in the treatment of obesity and sarcopenic obesity, despite the underlying multiorgan molecular mechanism is still not completely elucidated.

## Highlights

• BRD4 pharmacologic inhibition triggers rapid body weight loss.
• JQ1 induces adipose tissue depletion preserving skeletal muscle mass and strength.
• JQ1-induced adipose tissue loss does not alter systemic glucose and improves lipid homeostasis.
• JQ1 exerts a lipolytic action, inducing ATGL and HSL transcripts in the adipose tissue.
• In vitro, JQ1 reduces lipid accumulation and increases glycerol release in mature adipocytes.

## Graphical abstract

**Figure:**
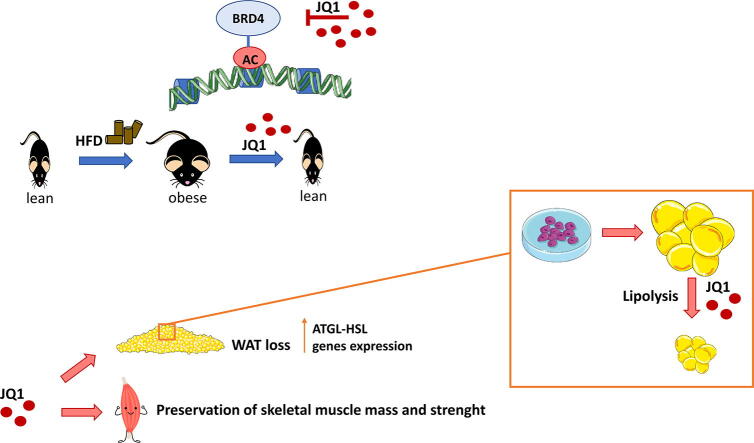

## Introduction

Obesity is a multifactorial chronic condition with a prevalence in middle-aged and older adults that continues to increase, representing one of the major public health problems in western countries [1]. Obesity, usually defined as BMI ⩾ 30 kg/m2, is characterized by an excessive fat accumulation that, occurring both in fat depots and ectopically, compromises physical function [1]. It is associated with an increased risk to develop other chronic diseases and is frequently associated with the presence of sarcopenia [1], [2]. This latter is nowadays considered an age-associated disorder, although it can appear earlier in life secondarily to several diseases, and is characterized by a progressive decline in skeletal muscle mass and functional capacity accompanied by an increased risk of adverse health-related outcomes such as physical disability, hospitalization, institutionalization, poor quality of life and mortality [3], [4]. Measurements of skeletal muscle mass and strength are the main criteria for the diagnosis of sarcopenia, although in the absence of specific guidelines, discriminating sarcopenic from non-sarcopenic individuals in the clinical practice is not an easy task [5], [6], [7]. Sarcopenia is very common among older adults with obesity, resulting in an emerging syndrome defined as sarcopenic obesity (SO) [2], [8], characterized by reduced skeletal muscle mass and function coupled with adipose tissue gain. The occurrence of SO is associated with metabolic complications, disability, morbidity and mortality which are significantly more important than those caused by sarcopenia or obesity alone, markedly raising healthcare costs [5], [9], [10], [11]. Age, sedentary lifestyle, genetics factors, nutrition, hormones, insulin resistance and diabetes represent risk factors for the onset of SO [12]. More specifically, this latter is characterized by a low-grade inflammatory status, mitochondrial dysfunction and oxidative stress together with fat infiltration into skeletal muscle [11], [12]. In aging sarcopenia, fat redistribution involves a reduction of subcutaneous fat, as well as an increase in visceral and intramuscular fat, lowering muscle quality and performance [13]. Understanding the underlying mechanisms of whole body fat control could lead to the identification of specific and selective targets for the treatment of SO.

In recent years, the bromo and extra-terminal domain (BET) family of proteins emerged as important epigenetic regulators of several cellular processes [13]. Selective BET inhibitors have been characterized as promising prospective therapeutic drugs for cancer, metabolic, inflammatory and autoimmune diseases [14], [15]. In mammals, there are four BET members, namely BRD2, BRD3, BRD4 and BRDT, characterized by two tandem bromodomains (BRDs), an extra terminal domain (ET) and a carboxy-terminal domain [15]. More specifically, BET proteins act as epigenetic readers by binding to acetylated lysine on histones and promoting gene transcription [16]. In particular, BRD4 has emerged as a coordinator of gene transcription in physiological and pathological processes [14]. It acts as a Ser2 kinase and phosphorylates the C terminal domain Serine 2 on RNA Pol II, stimulating RNA transcription [17]. Independently from gene transcription, BRD4 is involved in cell growth and cell cycle regulation, promoting the progression to S phase, while its knockdown results in G1 arrest [13], [16]. Among BET inhibitors, JQ1 is a thieno-triazolo-1,4-diazepine, a small cell-permeable molecule which binds competitively to the acetyl-lysine pocket of BRD4 with highest specificity and affinity, displacing BRD4 from chromatin and showing therapeutic effects in several diseases [13]. Recently, JQ1 was reported to modulate the inflammatory response, by reducing the recruitment of immune cells and downregulating the expression of pro-inflammatory cytokines and chemokines (including IL-6 and TNF-α) [13]. Recent studies showed that JQ1 was able to reduce oxidative stress, fibrosis and inflammation, improving skeletal muscle conditions and preserving muscle strength and integrity in murine models of cancer cachexia [18], Duchenne Muscular Dystrophy (DMD) [19], and in aging (unpublished observations). In parallel, a potential action of JQ1 in regulating lipid metabolism emerged [18], [20]. More specifically, in vitro experiments performed on differentiating 3 T3-L1 adipocytes revealed a JQ1-induced repression of adipogenesis [21]. Moreover, HepG2 hepatocytes exposed to JQ1 showed a significant decrease in intracellular lipid content, accompanied by a modulation of proteins involved in lipid biosynthesis, in particular decreased Acyl Coenzyme A carboxylase (ACC) and 3-hydroxy-3-methylglutaryl Coenzyme A reductase (HMGCR) protein expression [20]. Moving from these observations, and since no studies conducted so far elucidated the impact of JQ1 on lipid metabolism in mature adipose tissue, the present work was aimed to elucidate JQ1 role on fat metabolism in order to propose the epigenetic modulation as a potential target for treating obesity and SO.

## Materials and methods

### Animal model and experimental design

All experiments involving animals were conducted according to the ethical policies and procedures approved by the ethics committee of the University of Torino, Italy (authentication number 579/2018-PR). Experimental animals were cared for in compliance with the Italian Ministry of Health Guidelines and the Policy on Human Care and Use of Laboratory Animals (NRC, 2011). Despite epidemiological studies showing a prevalence of developing obesity in women, sex comparisons’ studies demonstrated different findings in rodents [22], [23]. In our study we decided to use C57BL/6J male mice because, according to the literature [22], [23], C57BL/6J mouse strain represent the most used animal model for diet-induced obesity (DIO) studies and, in particular male mice, are more susceptible to diet-induced weight gain and more prone to develop obesity and metabolic syndrome if fed with an HFD [22], [23]. Two experiments were performed, using three weeks old mice (Charles River Laboratories) that were randomized into subgroups and left in quarantine for one week. All animals were maintained on a regular dark-light cycle and received food and tap water ad libitum. In the first long-term experiment the mice were divided into four groups, two fed a high-fat diet (HFD; D12331, Sniff Spezialdiäten GmbH, Soest, Germany; composition: 26 % calories from carbohydrates, 59 % from fat, 15 % from proteins) and two fed a purified standard diet (SD; Sniff Spezialdiäten GmbH, Soest, Germany) for 22 weeks (long-term; [24], [25]. Over the last 14 days (from week 20), 10 mice/group received an intraperitoneal injection of JQ1 (20 mg/kg/day, dissolved in DMSO at the concentration of 35 mg/mL and diluted in (2–hydroxypropyl)-β-cyclodextrin 10 % in sterile saline solution) or vehicle in a volume of 100uL/injection. JQ1 dose and concentration were selected according to previous experiments, however, possibly due to the combination with the long-term exposure to HFD, the animals suffered from marked toxicity. For this reason, a second experiment was performed in which mice were fed the HFD for 12 weeks (mid-term), then were divided into two groups, respectively receiving for the last 14 days (from week 10) an intraperitoneal injection of 100uL of JQ1 at a dose of 10 mg/kg/day, dissolved in DMSO at the concentration of 45 mg/mL and diluted in (2–hydroxypropyl)-β-cyclodextrin 10 % in sterile saline solution (n = 11) and vehicle (n = 10). In the mid-term experiment in order to avoid the unexpected toxicity we saw in the long-term, JQ1 was dissolved at the limit of solubility in DMSO (45 mg/mL). Body weight and food intake were evaluated weekly. Fasting glycemia was measured by a glucometer the day before the beginning of JQ1 treatment and just before animal sacrifice by tail vein blood collection. At sacrifice, mice were anesthetized by isoflurane inhalation, the blood was collected by cardiac puncture, the plasma was isolated after centrifugation for 15 min at 1960 rcf and stored at − 80 °C. Several tissues were rapidly removed, weighed, frozen in liquid nitrogen and stored at − 80 °C for histological and biochemical analysis. The tibialis anterior (TA) muscle was weighed and frozen in isopentane for histological analysis.

### Grasping test

Voluntary muscle strength was evaluated by a grasping test at the beginning of JQ1 administration and the day before animal sacrifice. The mice were placed on a grid connected to a dynamometer, and animal force was assessed after gently pulling the tail. Three repetitions were performed for each mouse.

### Plasma analyses and lipid profile

#### Bio-plex multiplex immunoassay system

Cytokine (TNF-α, IL-1β, IL-6) levels were measured by using a Bio-Plex Multiplex Immunoassay System (Bio-Rad Laboratories, CA, USA). Plasma and tissue lipid profile was determined by measuring the content of triglycerides (TGs CL53-200S), total cholesterol (CL21-200S) and high-density-lipoprotein (HDL 7056) using commercial colorimetric kits (FAR DIAGNOSTICS, Verona, Italy). Briefly, plasma was assessed directly, while liver and quadriceps were homogenized in 5 % Triton X-100 (10 %w/v) using a bead homogenizer and subjected to two thermal shock cycles (on ice and at 95 °C) for 5 min each in order to extract the lipids. The supernatant was collected after 10 min centrifugation at 15,300 rcf and samples were processed following manufacturer instruction. All samples, blank and standards were prepared in duplicate.

#### Free fatty acid assay

Free fatty acid concentration in the plasma was quantified using a commercial kit (MAK044, Sigma), according to the manufacturer instructions. All samples, blank and standards were prepared in duplicate.

### Histological staining

#### Succinate dehydrogenase enzymatic activity and histological staining

Quadriceps muscles were homogenized (10 % w/v) using a bead homogenizer in ice-cold 150 mM NaCl, 10 mM KH_2_PO_4_, and 0.1 mM EGTA and centrifuged 5 min at 800 g. The supernatant was collected and 10 µL of protein homogenates were incubated with 200 µL reaction buffer containing 10 mM Na-succinate, 10 mM CaCl_2_, 2 mM KCN, 50 µg/ml DCPIP, 10 mM HEPES buffer (pH 7.4) and 0.05 % BSA. The absorbance (590 nm) was measured after 0-, 3-, 10- and 20-min incubation at 37 °C. All samples and blanks were prepared in duplicate and normalized quantifying the total protein content according to Bradford (BioRad, Hercules, CA, USA). The SDH staining was performed by incubating 10 µm-thick frozen sections of tibialis anterior for 10 min at 37 °C with a solution containing 1 mg/mL NTB (nitrotetrazolium blue chloride) and 27 mg/mL Na-succinate in PBS 1X. The whole TA section was digitally reconstructed by using the GIMP software.

### Protein expression analyses

#### Protein extraction

Cytosolic and nuclear extracts from gastrocnemius muscle were obtained by homogenization of 50 mg of tissue (5 % w/v) in an ice-cold solution containing 10 mM HEPES pH 7.5, 10 mM MgCl_2_, 5 mM KCL, 0.1 mM EDTA pH 8 0.1 % Triton X-100, adding at the time of use protease and phosphatase inhibitors. Samples were centrifuged at 3000 rcf for 5 min and the supernatant saved as cytosolic extract. The pellet was resuspended in 500uL (for 50 mg of initial tissue) of an ice-cold buffer containing 20 mM HEPES pH 7.9, 25 % glycerol, 500 mM NaCl, 1.5 mM MgCl_2_, 0.2 mM EDTA pH8, adding at the time of use protease and phosphatase inhibitors. Samples were incubated on ice for 30 min, vortexed every 10 min and the supernatants were collected after centrifugation at 3000 rcf for 5 min. Protein concentration was determined using the Bradford assay (BioRad, Hercules, CA, USA) with bovine serum albumin as standard.

#### Western blotting

The samples were resolved in Midi PROTEAN TGX Precast Gels (Bio-Rad, Hercules, CA) and then were transferred using the Trans-Blot Turbo Transfer System (Bio-Rad, Hercules, CA). The following antibodies were used: PDK4 (1:1000 Abcam), PDH (1:1000 Abcam), P-PDH (1:1000 Millipore), CPT1 (1:2000 ProteinTech), OXPHOS (1:1000 Abcam), TOM20 (1:1000 Abcam), PGC1α (1:1000 Millipore). GAPDH (1:20000 Sigma) and vinculin (1:2000 Santa Cruz) were used as loading reference. Proteins were detected using the Bio-Rad ChemiDoc and quantified by densitometry with the ImageLab software. Western blot plots for all the samples are available in a supplementary file.

#### Gene expression analyses

Total RNA was isolated from muscles (quadriceps and gastrocnemius) and white adipose tissue (epididymal) using TriReagent (Sigma Aldrich, St. Louis MO) and RNeasy Lipid Tissue Kit (Qiagen) respectively, following manufacturer instructions. RNA concentration was determined using a NeoDot (UV/Vis Microvolume Spectrophotometer, CliniScience). Total RNA (1 μg) was *retro*-transcribed to cDNA (complementary DNA) using the iScript cDNA synthesis kit (Bio-Rad, Hercules, CA). Transcript levels of target genes of interest were quantified by quantitative real-time PCR (qPCR) using a SyBR Green Master Mix (Bio-Rad, Hercules, CA) and normalized to the expression of the housekeeping genes β- actin and HPRT. All samples were run in duplicate and analyzed. Primer sequences were the following: β- actin (FW: CTGGCTCCTAGCACCATGAAGAT; RV: GGTGGACAGTGAGGCCAGGAT), HPRT (FW: GGCCAGACTTTGTTGGATTTG; RV: TGCGCTCATCTTAGGCTTTGT), ATGL (FW: CAACGCCACTCACATCTACGG; RV: TCACCAGGTTGAAGGAGGGAT), HSL (FW: GGCTCACAGTTACCATCTCACC; RV: GAGTACCTTGCTGTCCTGTCC), LPL (FW: GGACGGTAACGGGAATGTATG RV: ACGTTGTCTAGGGGGTACTTAAA), PGC1α (FW: GCAACATGCTCAAGCCAAAC; RV: TGCAGTTCCAGAGAGTTCCA), UCP3 (FW: CCTACGACATCATCAAGGAGAAGTT; RV: TCCAAAGGCAGAGACAAAGTGA).

#### Adipocyte cell culture

Subcultures of 3 T3-L1 cells were maintained according to ATCC instructions [26]. The cells were expanded in Dulbecco’s modified Eagle’s medium (DMEM) high glucose supplemented with 10 % Bovine Calf Serum (BCS) and penicillin/streptomycin (expansion medium) in 55 cm^2^ culture dishes, at 37 °C in a humidified atmosphere of 5 % CO_2_ in air until 70–80 % confluence is reached. Subsequently, cells were trypsinized and re-seeded (35000 cells/well) in 12 well plates. When 100 % confluence was reached, cells were further incubated for 48 h in the expansion medium, then shifted to the differentiation medium containing 1 µg/mL insulin, 1 µM dexamethasone, 0,5 mM IBMX and 10 % Fetal Bovine Serum (FBS). After further 48 h the differentiation medium was replaced with a maintenance medium completed with 1 µg/mL insulin and 10 % FBS, replaced every 48 h. Fully differentiated cells (14 days after induction) were subsequently treated with JQ1 (1 µM) for 8 days. Undifferentiated cells (maintained in the expansion medium) were used as negative controls. In some experiments, adipocytes were cultured in low glucose or low insulin medium, or low glucose-low insulin medium, or plasma mimicking medium (plasmax, Cancer Tools, UK), in the presence or in the absence of JQ1. The medium in all the experiments was changed and refreshed every two–three days in each well. All reagents were purchased from Sigma Chemical Company (St. Louis MO, USA).

#### Oil Red O staining

Differentiated adipocytes were fixed for 15 min in 4 % paraformaldehyde (PAF), washed twice in PBS, completely dried and incubated for 20 min in Oil Red O. A stock solution was prepared by dissolving 0,5% Oil Red O (w/v) in isopropanol and then filtered through a 0.2-μm filter. Subsequently, a fresh Oil Red O working solution was prepared by mixing the stock solution with distilled water to reach a final concentration of 65 % isopropanol. Microscope images were taken after the solution was removed and wells washed, allowing to observe the lipid droplets in differentiated adipocytes. In order to quantify the staining, an extraction solution (ethanol:diethyl ether 4:1) was added to each well and incubated for 15 min, reading the absorbance at 490 nm. All samples were prepared in triplicate.

#### Glycerol assay

Glycerol concentration was determined by a coupled enzyme assay (MAK117, Sigma-Aldrich) based on glycerol kinase and glycerol phosphate oxidase, resulting in a colorimetric (570 nm) product, proportional to the glycerol present, according to the manufacturer instructions. 3 T3-L1 differentiated adipocytes were incubated in a medium free of phenol red that was collected after 2-, 4-, 6- and 8 days. All samples, blank and standards were prepared in duplicate.

## Results

### JQ1 induces adipose tissue wasting without affecting skeletal muscle mass and function

**Fig. 1:**
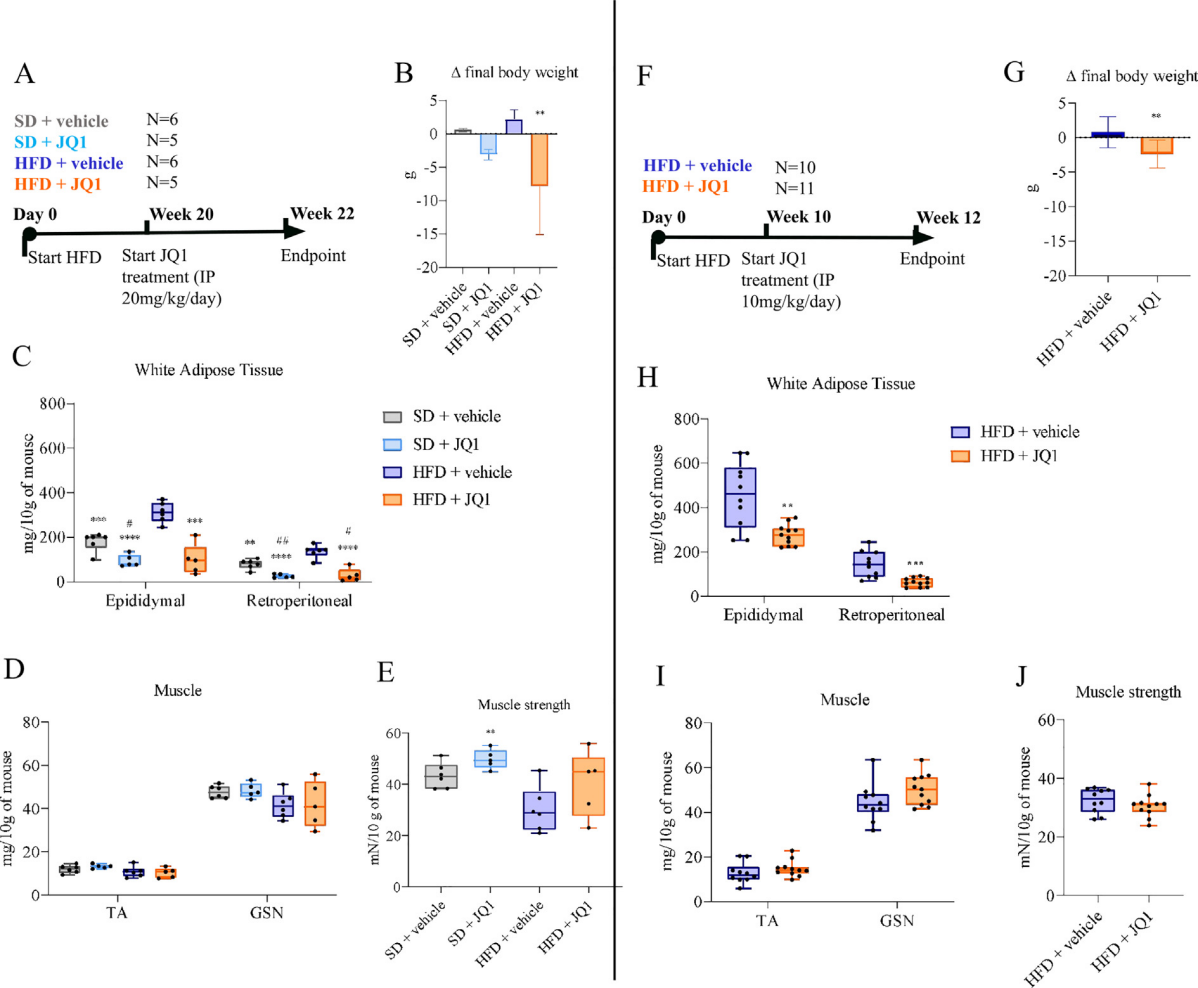
JQ1 induces adipose tissue wasting without affecting skeletal muscle mass and function. Timeline and schedule of (A) long-term experiment: mice were exposed for 20 weeks to high fat diet (HFD) or standard diet (SD) and then treated with 20 mg/kg/day JQI or vehicle for 2 weeks, and (F) mid-term experiment: mice were exposed for 10 weeks to HFD or SD and then treated with 10 mg/kg/day JQ1 or vehicle for 2 weeks. Differences in body weight (B, G), epididymal and retroperitoneal white adipose tissue (C, H), tibialis anterior (TA) and gastrocnemius (GSN) muscles (D. I) and relative muscle strength (E, J). Tissue weight and muscle strength are expressed in milligrams and unit force (mN), respectively, and normalized for 10 g of body weight. Data are means +/- SD of 5–6 mice per group in the long-term experiment, 10–11 mice per group in the mid-term experiment. Statistical significance: #p <.05, ##p <.01, ###p <.001 vs SD + vehicle; *p <.05, **p <.01, ***p <.001 vs HFD + vehicle.

**Supplementary figure 1:**
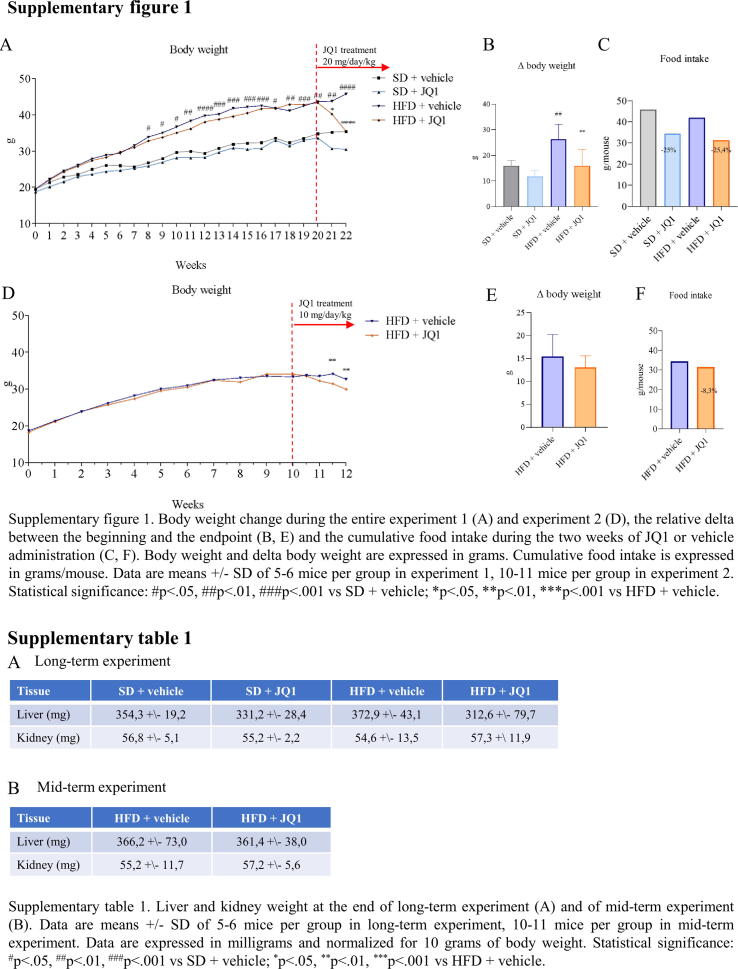


Two independent experiments lasting, respectively, 22 (long-term) or 12 (mid-term) weeks, were performed exploiting a diet-induced obesity (DIO) model in order to mimic severe and mild metabolic syndrome and to evaluate the impact of JQ1 on adipose tissue homeostasis (See Fig. S1A/D, B/E for body weight kinetik). In the long-term experiment, schematically explained in Fig. 1A, we observed a significant body weight loss after JQ1 administration in both SD and HFD fed mice, especially in HFD + JQ1 group compared to HFD + vehicle group (Fig. 1B and S1 A). Such a pattern is associated with a significant reduction in epididymal and retroperitoneal adipose tissue mass (Fig. 1C), supporting the hypothesis that JQ1-induced body weight loss occurs targeting the adipose tissue. Conversely, lean mass and in particular the skeletal muscle weight was not affected by neither the HFD regimen nor by the JQ1 treatment (Fig. 1D), although a tendency towards lower muscularity in both HFD groups was observed, mimicking sarcopenic obesity. In order to better understand the impact on the skeletal muscle, the assessment of muscle strength showed an increase in JQ1-treated mice fed with the SD, while only a trend to increase could be observed in the HFD + JQ1 group, again with no statistical differences between SD and HFD groups (Fig. 1E), not allowing to reproduce an overt condition of sarcopenic obesity.

The long-term schedule, however, resulted in high blood aspartate transaminase (AST) levels and in reduced animal survival, suggesting the occurrence of HFD interference on JQ1 tolerance. For this reason, we performed a second experiment aiming to achieve less metabolically compromised animals (mid-term schedule): HFD exposure was reduced to 12 weeks and JQ1 dose to 10 mg/kg/day (Fig. 1F). As in the first experiment, JQ1 prompted a significant body weight loss (Fig. 1G, and Figure S 1D), accompanied by significant reduction of adipose tissue mass (Fig. 1H) and by preserved muscle weight (Fig. 1I) and function (Fig. 1J). In order to ascertain whether the differences in body weight observed in JQ1-treated mice were due to a different feeding behavior, the cumulative food intake was assessed, showing that, even if JQ1 was able to slightly reduce food consumption (Figure S1 C, F), such decrease likely marginally contributes to the observed phenotype. Both HFD and JQ1 were unable to modify liver and kidney mass (Table S1 A-B).

### JQ1-induced adipose tissue loss improves systemic lipid homeostasis

**Fig. 2:**
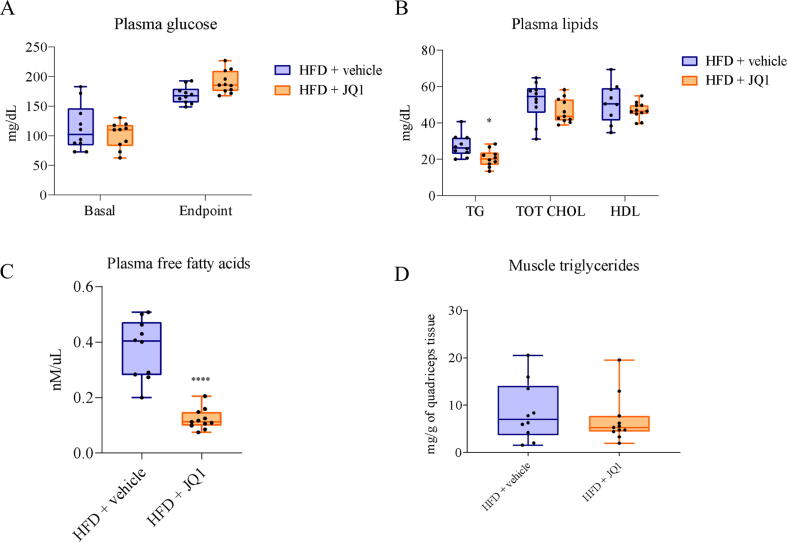
JQ1 does not alter glucose and lipid levels, but reduces plasmatic free fatty acids. Evaluation of glucose (A), total (TOT CHOL) and HDL cholesterol (B), free fatty acids (C) in the plasma of mice exposed for 10 weeks to HFD and then treated with 10 mg/kg/day JQ1 for 2 weeks. Triglycerides are evaluated both in plasma (B) and muscle (D) and expressed respectively in milligrams per deciliter and in milligrams per gram of tissue. Data are means +/- SD of 10–11 mice per group. Statistical significance: * p < 0.05, ** p < 0.01 *** p < 0.001 vs HFD + vehicle.

The rapid loss of adipose tissue induced by JQ1 might result in a massive release and accumulation of fatty acids in the circulation, altering systemic glucose and lipid homeostasis, and consequently the liver, which is the organ that mainly controls metabolic fluxes. In the long-term experiment, no differences in plasma glucose, TGs, total and HDL cholesterol levels were observed after JQ1 administration compared to the vehicle-treated groups (Table 1A). Such a pattern could reflect lipid over-accumulation in non-adipose tissues, such as the liver. However, the amount of hepatic TGs, total and HDL cholesterol remained comparable among the different experimental groups (Table 1A). Obesity and metabolic syndrome frequently trigger a systemic inflammatory response [27], [28]; consistently, the analysis of circulating pro-inflammatory cytokines (TNF-α, IL-6, IL-17) demonstrated that HFD exposure increased TNF-α levels and that such an increase could be counteracted by JQ1 (Table 1B). As for the mid-term experiment, while no changes could be observed in glycemia, both basal and at the end-point (Fig. 2A), a significant reduction was observed in TG levels in JQ1-treated mice (Fig. 2B). Despite the lack of SD fed mice in the mid-term experiment design, TG reduction upon JQ1 administration results in TG levels similar to the SD fed mice of the long-term experiment. Similarly to the long-term schedule, both total and HDL cholesterol plasma levels were unaffected by JQ1 (Fig. 2B). TG reduction in JQ1-treated mice was paralleled by a marked decrease of plasma FFA (Fig. 2C), while no TG accumulation was observed in the skeletal muscle (Fig. 2D), suggesting that the fatty acids released from the adipose tissue upon JQ1 treatment are actively consumed rather than accumulated.

**Table 1:** JQI does not affect systemic and tissue metabolic and inflammatory profile in the long-term experiment.

**A**				
**Tissue and systemic lipid profile**	**SD + vehicle**	**SD + JQ1**	**HFD + vehicle**	**HFD + JQ1**
**Plasma**				
Glucose (basal) (mg/dL)	85.8 +/- 17.9	72.8 +/- 14.2	108.8 +/- 22.7	109.5 +/- 19.5
Glucose (endpoint) (mg/dL)	146.7 +/- 21.7	190.8 +/- 29.5	180.8 +/- 15.5	150 +/- 51.1
TGs (mg/dL)	21.7 +\- 6.3	26.2 +/- 7.5	25.9 +/- 8.8	25.6 +/- 2.6
total cholesterol (mg/dL)	73.0 +/- 10.1	70.9 +/- 12.3	88.5 +/- 15.8	73.3+/- 26.3
HDL cholesterol (mg/dL)	57.0 +\- 6.4	62.1 +/- 18.0	63.7 +/- 6.8	67.4 +/- 49.6
**Liver**				
TGs (mg/g)	263.4 +/- 84	338.5 +/- 44.8	313.4 +/- 135.3	301.3 +/- 173
total cholesterol (mg/g)	15.7 +/- 3	15 +/- 1.8	13.2 +/- 2.1	13.3 +/- 5.3
HDL cholesterol (mg/g)	14.9 +/- 5.3	14.3 +/- 0.8	11.3 +/- 2.6	11.7 +/- 3.2
B				
**Inflammatory profile**	**SD + vehicle**	**SD + JQ1**	**HFD + vehicle**	**HFD + JQ1**
TNF-α (pg/mL)	9.4 +/- 1.5	7.1 +/- 4.5	19.6 +/- 5.3 ##	10.4 +/- 5.5 *
IL-6 (pg/mL)	37.9 +/- 15.8	14.9 +/- 3.8	54.2 +/- 24.5	34.2 +/- 16.5
IL-17 (pg/mL)	132.3 +/- 114.3	43.1 +/- 22.1	138.2 +/- 49.6	68.1 +/- 6.0

Tissue and systemic metabolic profile (A), analyzed in mice exposed for 20 weeks to HFD and then treated with 20 mg/kg/day JQ1 for 2 weeks. Inflammatory cytokine profile (B) evaluated in plasma, in particular TNF-a, IL-6 and IL-17 are reported. Data are means +/- SD of 5–6 mice per group. Statistical significance: \# p < 0.05. \# ip < 0.01.###p <.001 vs SD + vehicle; *p <.05, **p <.01, ***p <.001 vs HFD + vehicle.

### Impact of JQ1 on oxidative capacity in muscle and liver

**Fig. 3:**
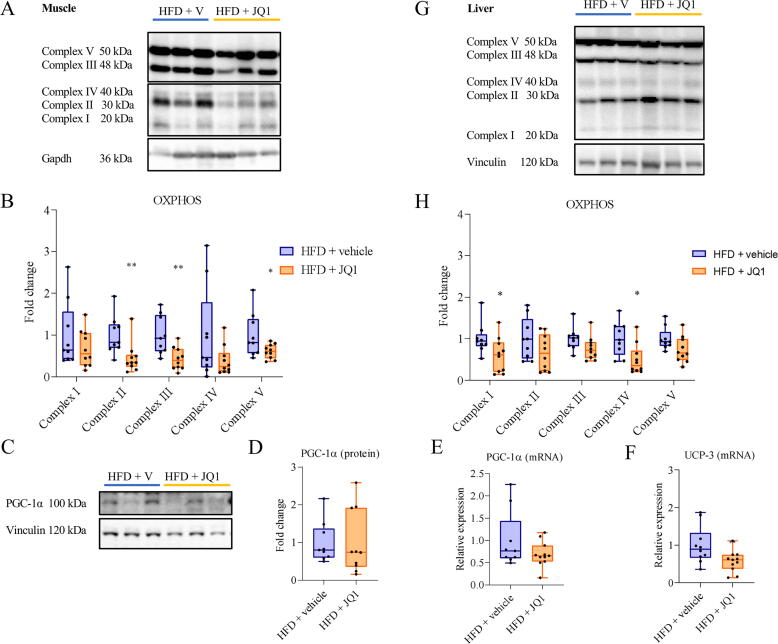
JQ1 does not affect muscle and liver oxidative capacity. Representative immunoblotting showing the expression of complexes of mitochondrial respiratory chain and PGC-la in skeletal muscle (A, C) and liver (G) in the mid-term experiment. Densitometric analysis is normalized for the corresponding vinculin or gapdh content and expressed as fold change relative to HFD + vehicle (B, D, H). PGC-10 and UCP-3 gene expression in skeletal muscle (E, F). Data are means +/- SD of 10–11 mice per group. Statistical significance: * p < 0.05 **p < 0.01 ***p <.001 vs HFD + vehicle.

**Supplementary figure 2:**
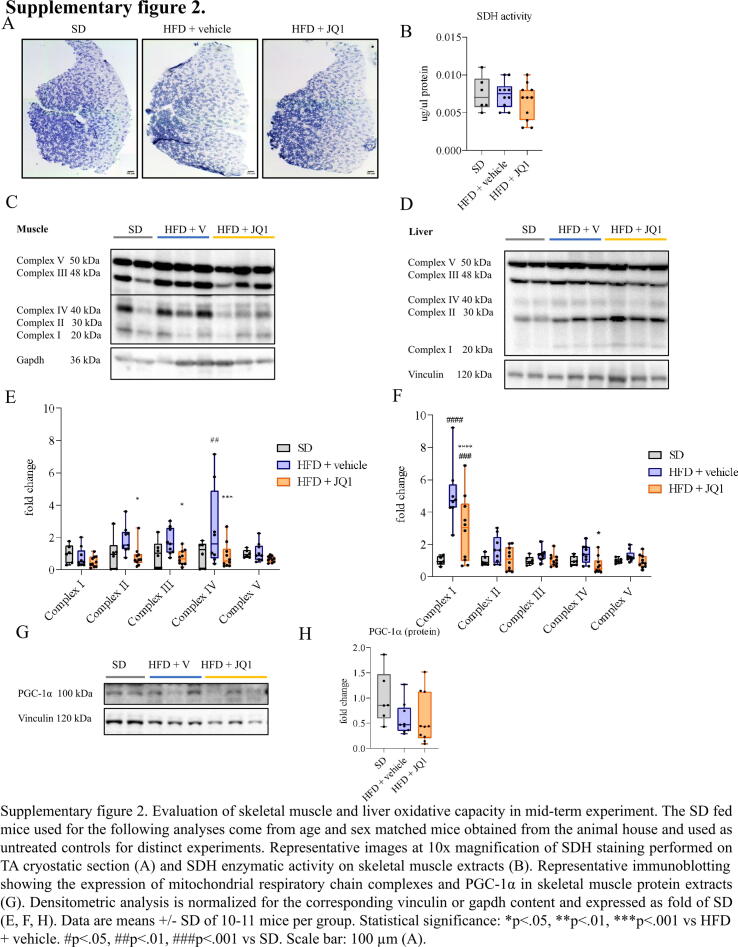


The lack of mobilized lipid accumulation in the circulation and in the tissues of HFD + JQ1 treated mice could potentially result from enhanced oxidative capacity. In the skeletal muscle of animals of the mid-term experiment, the HFD-induced adaptation previously shown to induce muscle accumulation of OxPhos proteins [29] and Figure S2 C, E, was partially reversed by treatment with JQ1 that was able to reduce the expression of complexes II, III and V, without changing the expression of both complexes I and IV (Fig. 3A, B). Both protein and gene expression of the master regulator of mitochondrial biogenesis, PGC-1α [30] were comparable in HFD exposed mice, in the presence or in the absence of JQ1 (Fig. 3C-E). To have an insight into the possibility that JQ1-induced lipid mobilization could fuel thermogenesis, we evaluated UCP-3 [31], [32] gene expression in the skeletal muscle. The results exclude such an option, since UCP-3 transcript levels were unchanged, or even tended to decrease, in JQ1-treated and untreated mice (Fig. 3F). In line with these data, succinate dehydrogenase (SDH) staining and enzymatic activity in the TA muscle showed no differences in JQ1-treated mice compared to controls (Fig. S2A, B).

As for the liver, protein expression of OxPhos was evaluated, observing that JQ1 treatment was able to reduce the expression of complexes I and IV only, leaving unaltered the expression of the other complexes (Fig. 3G, H, and Figure S2 D, F).

**Supplementary figure 3:**
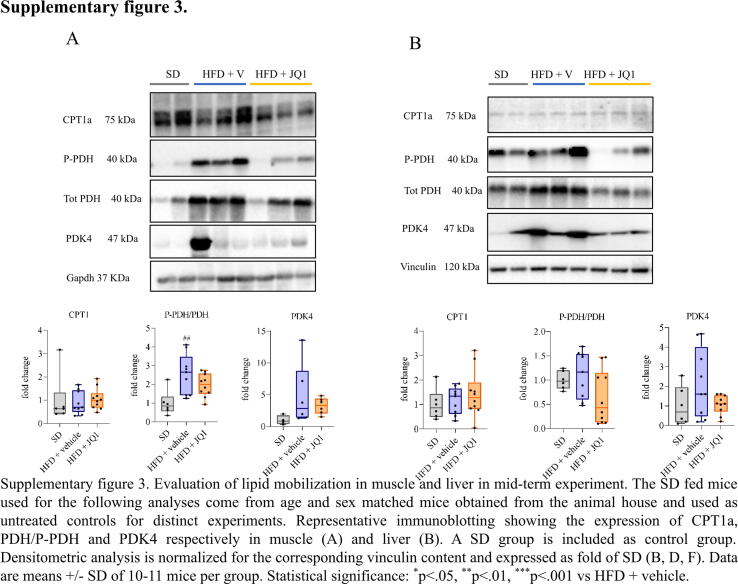


The reduced circulating FFA reported above could result from increased fatty acid beta-oxidation (FAO) in both liver and muscle. For this reason, we analyzed the protein expression of carnitine palmitoyl transferase 1a (CPT1a), the key regulatory enzyme in FAO involved in the import of long-chain fatty acids into mitochondria, together with the pyruvate dehydrogenase complex (PDH), responsible for the conversion of pyruvate into acetyl-coenzyme A and its inhibitor, the pyruvate dehydrogenase kinase 4 (PDK4), critical for regulating cellular energy metabolism [33], [34], [35]. However, no significant differences among the groups were observed in either tissue (Fig. S3A, B).

### JQ1 stimulates lipolysis in the adipose tissue

**Fig. 4:**
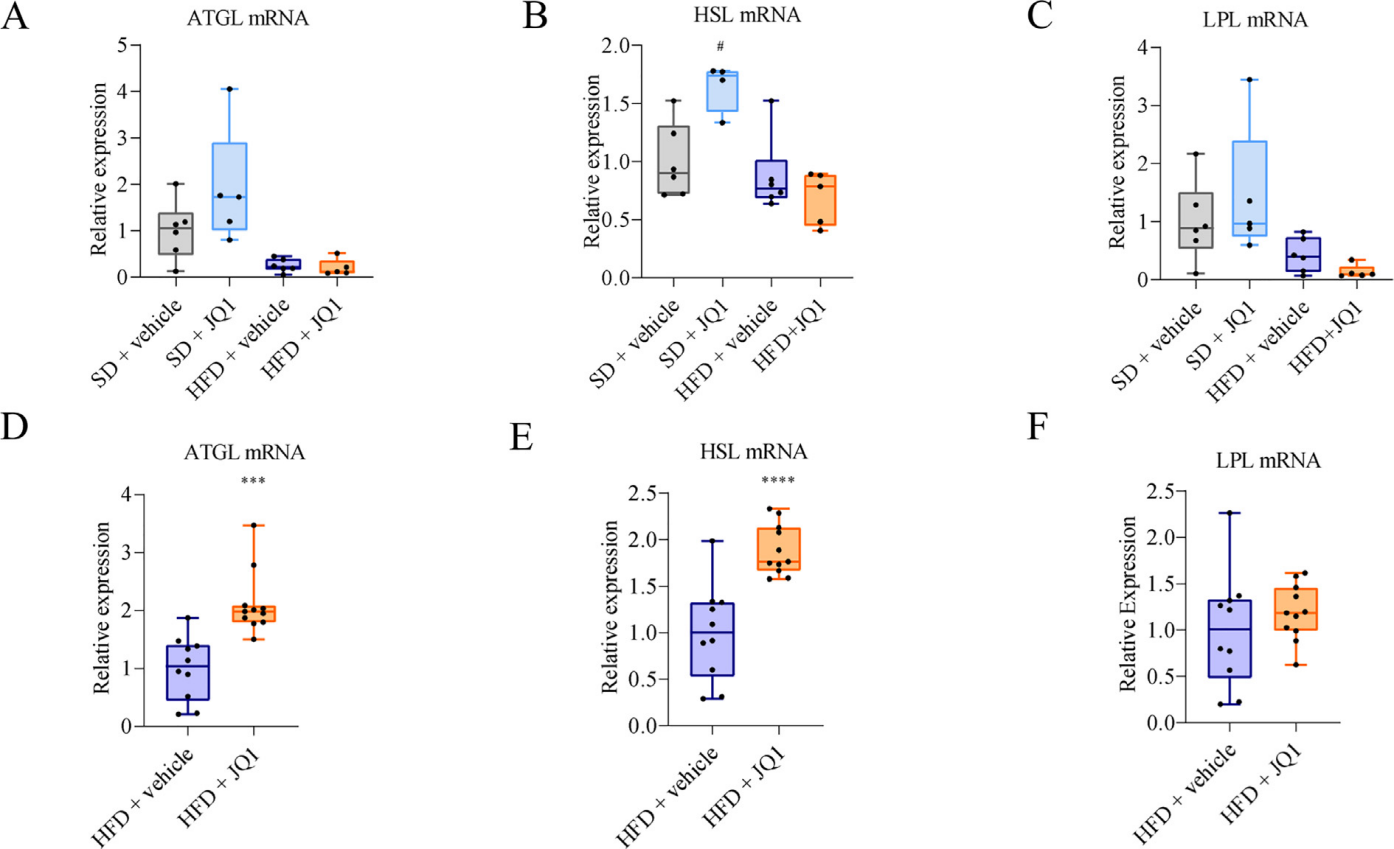
JQ1 stimulates the expression of genes involved in lipolysis in the adipose tissue. PCR analysis for ATGL (A-D), HSL (B-E) and LPL (C-F) in adipose tissue mRNA extracts. Data are means +/- SD of 5–6 mice per group in experiment 1, 10–11 mice per group in experiment 2. Statistical significance: ## p < 0.05, p <.01, ###p <.001 vs SD + vehicle; therefore p < 0.05^* p < 0.01. **p <.001 vs HFD + vehicle.

To investigate the mechanism by which JQ1 impinges on the adipose tissue, transcript levels of genes involved in lipolysis were analyzed. Many are the enzymes involved in this pathway: adipose triglyceride lipase (ATGL), responsible for the first and rate-limiting step, hormone-sensitive lipase (HSL), involved in releasing FFA from adipose tissue to circulation and lipoprotein lipase (LPL), implicated in lipid uptake from the circulation [36], [37], [38], [39], [40]. In the long-term experiment JQ1 treatment was able to increase the expression of HSL only in animals fed the standard diet (Fig. 4A, C), possibly due to the late stage chronic condition of the animals that could have made the adipose tissue insensitive to the stimulation. On the contrary, in the mid-term experiment, JQ1 was able to upregulate ATGL and HSL expression, but not LPL (Fig. 4D, F), suggesting a sustained JQ1 lipolytic action.

### JQ1 induces lipolysis in vitro

**Fig. 5:**
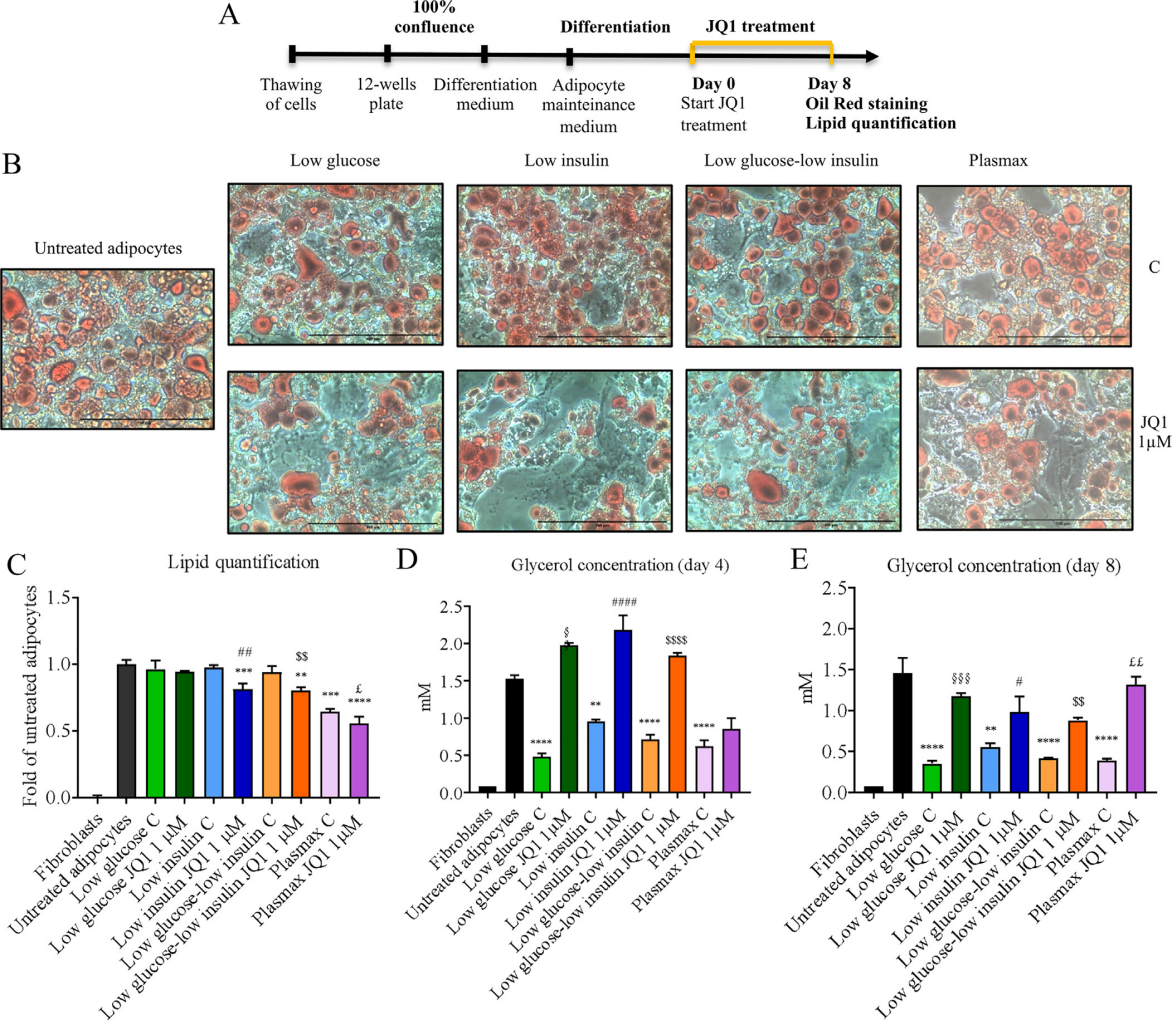
JQ1 directly stimulates lipolysis and glycerol release in 3 T3-L1 cells. Representative images of Oil Red O staining performed on 3 T3-L1 cells treated after complete differentiation with 1 µM JQ1 for 8 days in low glucose, low insulin, low glucose + low insulin condition or Plasmax (B). Lipid quantification is expressed as fold of untreated adipocytes (C). 3 T3-L1 glycerol release in cell medium after 4 days (D) and 8 days (E) from the beginning of JQ1 treatment. Data are means +/- SD. Statistical significance: * p < 0.05, ** p < 0.01, *** p < 0.001 neg****D < 0 vs untreated adipocytes; ^§^p < 0.05 ^§§^p < 0.01 ^§§§^ p < 0.001 ^§§§§^p < 0.0001 vs low glucose C; ^#^p <.05, ^##^ p < 0.01 ^###^ p < 0.001 ^####^p <.001 vs low insulin C; ^$^p < 0.05, ^$$^p <.01, ^$$$^p <.001, ^$$$$^p <.001 vs low glucose-low insulin C; ^£^p <.05, ^££^p <.01, ^£££^p <.001, ^££££^p <.001 vs plasmax C; Scale bar: 100 µm (B).

**Supplementary figure 4:**
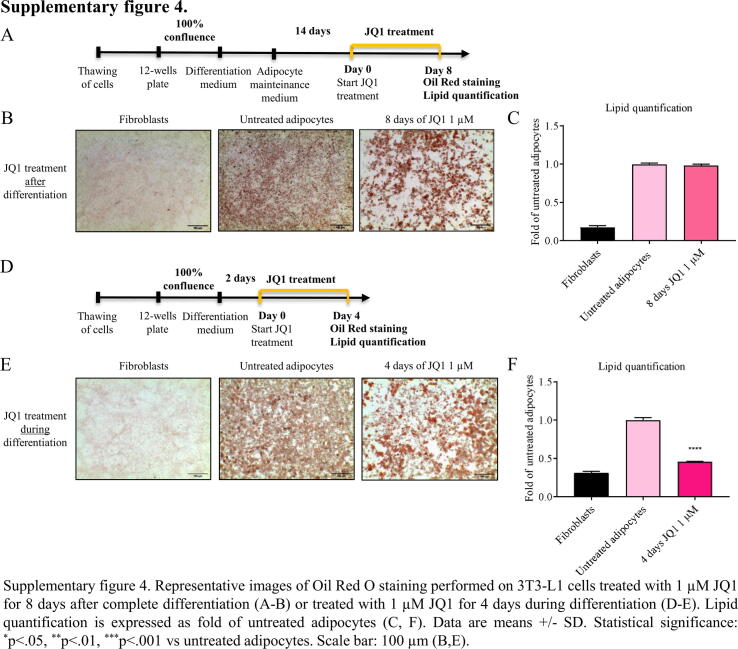


In order to ascertain that JQ1 is able to directly induce lipolysis in the adipocytes, 3 T3-L1 cells were terminally differentiated and treated with JQ1 for 8 days, as depicted in Fig. S4A. Lipid quantification showed no differences in lipid accumulation, in JQ1 vs untreated adipocytes (Fig. S4B, C). In order to check cell susceptibility to JQ1, we repeated the experiment exposing 3T3-L1 cells to JQ1 (4 days) during differentiation (Fig. S4D), according to [41]. The results show that indeed, JQ1 impairs the formation of lipid stores during adipocyte differentiation (Fig. S4E). However, this result has limited translational value, given that is insufficient to explain the in vivo JQ1 action on mature adipocytes. In the attempt to test JQ1 in a more physiological culture settings, fully differentiated 3T3-L1 cells were cultured in low glucose or low insulin media, or in the combination of both low glucose-low insulin or in the Plasmax medium (a particular medium that mimics the physiological and metabolic profile of human plasma) (Fig. 5A). The treatment with JQ1 for 8 days induced a significant reduction of lipid accumulation in low insulin, low glucose-low insulin and Plasmax conditions, the latter showing already lower basal lipid accumulation (Fig. 5B), compared to the relative untreated controls and also to the untreated adipocytes, as confirmed by neutral lipid quantification (Fig. 5C). In parallel, in order to estimate triglyceride catabolism, glycerol release into the medium was measured. Consistently with the reduced lipid accumulation, glycerol concentration in the spent media was elevated after 4 (Fig. 5D) and 8 (Fig. 5E) days of JQ1 exposure.

## Discussion

The BET protein inhibitor JQ1 is known to attenuate inflammation, oxidative stress, fibrosis and in general, to improve the skeletal muscle status in experimental cancer cachexia [18], DMD [19] and sarcopenia of aging (unpublished data). Moving from these studies, a serendipic observation highlighted a potential effect of JQ1 on the adipose tissue [18], [20]. In parallel to previously published works and given the emerging relationship between epigenetic alterations and disorders characterized by dysregulated lipid metabolism [20], [21], the aim of the present study was to clarify the impact of JQ1 on adipose tissue homeostasis in order to identify new targets for the treatment of obesity, including sarcopenic obesity. The results showed consistent and comparable loss of body weight and fat mass in either mid- or long-term HFD exposure, confirming a novel effect of JQ1 on adipose tissue, without impacting on skeletal muscle mass and strength.

Over the last years, the adipose tissue emerged as a key player involved in many physiologic and pathologic pathways, and no longer considered as a simple energy storage [42], [43]. In particular, the adipose tissue is recognized as a major endocrine organ involved in the production and the secretion of a large variety of pro and anti-inflammatory factors, including cytokines, chemokines and adipokines [42], [43].

The consistent loss of adipose tissue likely results from increased lipolysis, the biochemical process implicated in the catabolism of triglycerides (TGs) stored in cellular lipid droplets, generating FFA and glycerol [36], [38]. Enhanced lipolytic rates could lead to massive glycerol and FFA release into the circulation, on one side supplying to energetic demands, on the other resulting in increased FFA systemic levels, potentially triggering inflammation and deleterious redistribution of excess circulating lipids toward storage in non-adipose tissues, such as liver and muscle [37], [44]. The results presented in this study are in favor of a healthy weight loss, since no relevant differences in the inflammatory and lipid profiles could be attributed to JQ1 treatment or even in the long-term experimental conditions JQ1 was able to counteract the increase of TNF-α induced by HFD. These data suggest that, although JQ1 induces a rapid and massive adipose tissue loss, it does not aggravate systemic inflammation and lipid homeostasis, eventually improving them. It is important to state that on the one side that the metabolic syndrome associated with HFD regimens generally associates with increased inflammation, thus even inducing increased TNFa levels in the co-presence of a morbid overweight condition. The other way around, TNFa induces lipolysis and fat wasting in those pathological conditions usually associated with skeletal muscle wasting, such as cachexia, a completely different pathological state. We should thus consider JQ1-induced lipolysis and reduced TNFa circulating levels as two processes not necessarily conflicting. In our study JQ1 was able on one side to directly induce lipolysis independently from TNFa, parallelly preserving skeletal muscle mass and function, such an effect being in line with reduced TNFa circulating levels. The significant plasma FFA decrease suggests a possible fatty acid increased consumption induced by JQ1 administration. DIO represents one of the causes of muscle mass loss and impaired functionality [44]. An excessive lipid flux in the skeletal muscle, mainly due to exceeding the storage capability of adipose tissue, results in ectopic lipid deposition and fatty infiltration, which in turn generates lipotoxic stress [44], [45], [46]. In parallel, lipotoxicity is strongly associated with a marked impairment of mitochondrial biogenesis and fatty acid oxidation capacity, further amplifying lipid accumulation in the skeletal muscle [46], [47]. In the current study, the absence of skeletal muscle lipid infiltration as well as the unchanged TG content rule out the increase of intramuscular fat infiltration. The preservation of muscle mass and strength, accompanied by no differences in TG accumulation into the skeletal muscle and together with the reduction of circulating FFA, led to hypothesize a potential role for the muscle in consuming fats released by the adipose tissue upon JQ1 administration. In particular, the stimulation of lipolysis, thus reducing fat storage, accompanied in parallel by the oxidation of the newly released fatty acid, represents a healthy strategy to combat obesity. To test this hypothesis, we evaluated if JQ1 administration resulted in improved skeletal muscle mitochondrial status and oxidative capacity, which could be actively fueled by FFA, keeping in mind that many studies regarding skeletal muscle oxidative capacity in diet-induced obesity models yielded controversial results. Heo J. and colleagues demonstrated in metabolic diseases a reduction of mitochondrial content and oxidative capacity together with an increase in intramyocellular TG levels [48]. Obesity, high-fat diet feeding, as well as aging and low levels of physical activity may favor a reduction of PGC-1alpha expression and/or function in skeletal muscle, reducing OXPHOS complexes expression, contributing to insulin resistance and diabetes [48], [49]. On the contrary, Pinho R et al. observed an adaptive response to lipid oversupply characterized by an enhancement of mitochondrial biogenesis and oxidative capacity [50]. Moreover, Turner et al reported increased mitochondrial respiratory chain subunit protein expression and fat oxidation capacity in the skeletal muscle of rats fed a high-fat diet [51]. The lack of changes in SDH staining and activity and in PGC-1alpha protein and mRNA levels suggested that mitochondrial oxidative capacity, mass and biogenesis were not affected by JQ1 treatment. However, JQ1 was able to reduce to physiological levels the protein expression of mitochondrial respiratory chain complexes II, III and IV. These data were supported by no differences in the mRNA expression of UCP3, a regulator of lipid oxidation and thermogenesis in the skeletal muscle [31], [32]. In particular UCP3, characteristically expressed in skeletal muscle, is upregulated during all those conditions in which fatty acid supply exceeds oxidative capacity, such as high-fat diet consumption [32], [52]. On the contrary, during endurance training or weight loss, the metabolic efficiency is increased, followed by UCP3 down-regulation [32], [52]. Parallelly, fatty acid oxidation is mediated by pyruvate dehydrogenase (PDH), an enzyme complex linking glycolysis with oxidation, whose activity in the skeletal muscle results strictly regulated by pyruvate dehydrogenase kinase 4 (PDK4) [53]. Previous studies demonstrated after 3 days of HFD a substantial increase of PDK4 mRNA levels in the skeletal muscle, accompanied by an increase in carnitine palmitoyltransferase 1 (CPT1), a key enzyme responsible to the carnitine-shuttle import of long-chain fatty acids into mitochondria [53]. Moreover, adult skeletal muscle is characterized by the ability to adapt to a variety of external signals, such as the release of FFA from adipose tissue, switching easily between glucose and fat oxidation [38], [54], [55]. A sustained fatty acid import to mitochondria together with PDK4-mediated suppression of PDH activity induced by chronic lipid oversupply is able to inhibit mitochondrial glucose utilization, and could suggest a switch from carbohydrate to fatty acid oxidation (FAO) [37], [53], [55], [56]. For this reason, regulatory enzymes involved in FAO were analyzed in both skeletal muscle and liver, unfortunately not producing data in favor of a metabolic shift in such direction.

In order to investigate whether the reduced adiposity observed in vivo was due to lipolysis, transcript levels of lipases were analyzed in epididymal adipose tissue. In particular, a decrease in the expression of ATGL, the first enzyme involved in lipolysis and expressed predominantly in adipocytes, was previously reported in white adipose tissue of genetically obese mice [57], [58], [59]. Not surprisingly, ATGL low levels are able to reduce in a significant manner TAG hydrolysis in adipose tissue, resulting in TAG accumulation into adipocytes, and leading finally to obesity [58], [60]. Coherently, many studies evidenced a strong association between HSL impaired lipolytic activity and obesity [61]. ATGL and HSL mRNA levels resulted statistically increased in JQ1-treated mice. These results prompted us to test the occurrence of a direct lipolytic action of JQ1 on a cellular model of mature adipocytes. Given that the JQ1-induced in vivo phenotype has not been observed in vitro in high glucose cultured cells, we decided to adopt more physiological culture conditions, highlighting a stronger JQ1 effect especially for a combination of low insulin - low glucose or the use of a medium closer to the human serum, such as the Plasmax. According to the increased glycerol release into the medium, our data demonstrated a direct effect of JQ1 on lipolysis, likely contributing to the phenotype observed in vivo.

## Conclusion

In conclusion, our findings describe a novel effect of the BET protein inhibitor JQ1 in selectively decreasing white adipose tissue mass without impacting on skeletal muscle mass and strength or altering the systemic metabolic and inflammatory homeostasis. Despite deeper analyses to decipher the pathways responsible for JQ1 regulation of adipose tissue metabolism are still required, the data collected so far highlight the impact of JQ1-induced BET inhibition in improving the health status by synergistically acting on distinct organs, from adipose tissue to muscle, and maybe others that are still underexplored, suggesting a JQ1 potential benefit in the treatment of obesity.

## CRediT authorship contribution statement

**C laudia F ornelli:** Methodology, Writing - Original Draft, Writing - Review & Editing , Investigation, Visualization. **A lessia S ofia Cen:** Methodology, Writing - Review & Editing, Investigation, Visualization. L orenzo Nevi: Investigation and Visualization. Raffaella Mastrocola: Investigation and Visualization. **Gustavo Ferreira Alves:** Investigation and Visualization. **Paola Costelli:** Conceptualization, Writing - Review & Editing. **Giuseppina Caretti:** Conceptualization, Writing - Review & Editing. **Massimo Collino:** Conceptualization, Writing - Review & Editing. **Fabio Penna:** Conceptualization,Methodology, Writing - Review & Editing, Supervision and Funding acquisition

## Declaration of competing interest

The authors declare that they have no known competing financial interests or personal relationships that could have appeared to influence the work reported in this paper.

## Figures and Tables

**Supplementary figure 5:**
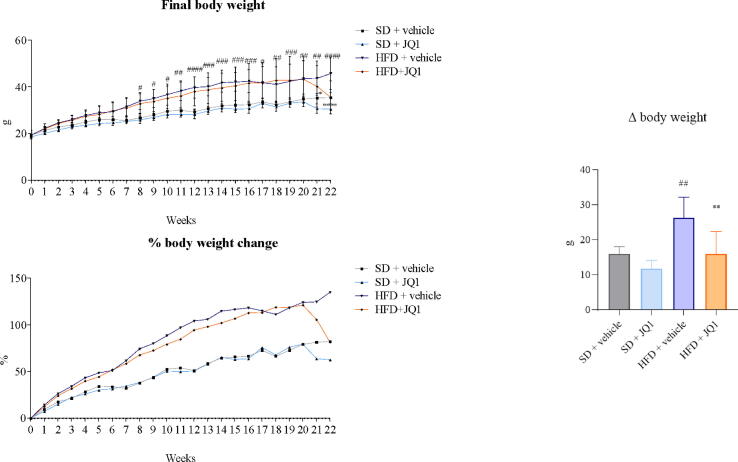


**Supplementary figure 6:**
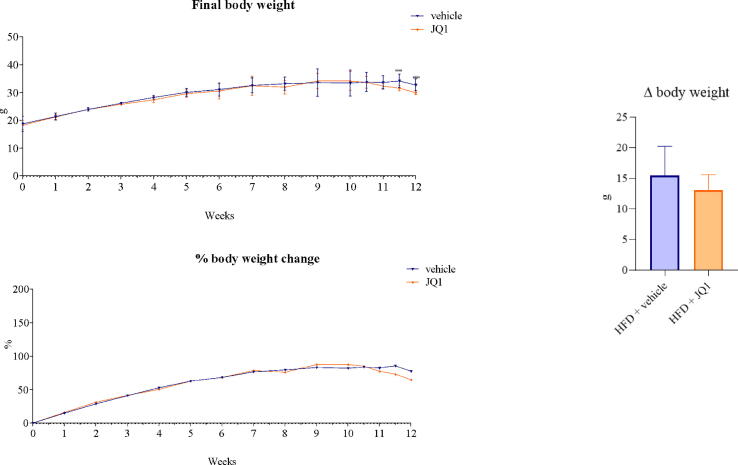


**Supplementary figure 7:**
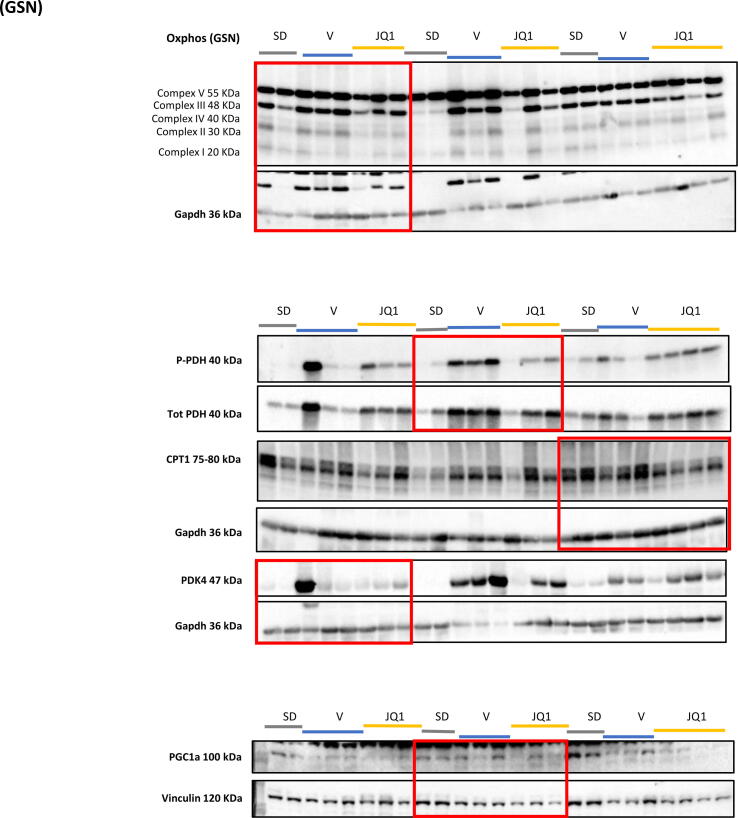


**Supplementary figure 8:**